# Editorial: Emerging horizons of metformin: exploring recent advances and addressing challenges in research and clinical utilization

**DOI:** 10.3389/fphar.2025.1674641

**Published:** 2025-08-28

**Authors:** Charupong Saengboonmee, Mehrnaz Abbasi, Agnieszka Śliwińska

**Affiliations:** ^1^ Department of Biochemistry, Faculty of Medicine, Khon Kaen University, Khon Kaen, Thailand; ^2^ College of Human Sciences, Auburn University, Auburn, AL, United States; ^3^ Department of Nucleic Acid Biochemistry, Faculty of Medicine, Medical University of Lodz, Lodz, Poland

**Keywords:** anti-diabetes, biguanide, drug repurposing, metformin, drug repositioning

Drug repurposing, or drug repositioning, is a popular strategy in drug development that aims to accelerate the process and reduce costs associated with discovering new drugs. (Tanoli et al., 2025). Metformin is an FDA-approved medication for type 2 diabetes that is also being researched for other potential uses (Saengboonmee et al., 2021; Foretz et al., 2023). Metformin has been shown to have multiple beneficial effects on various non-communicable diseases, particularly those related to metabolic dysregulation, such as obesity (Abbasi et al., 2022; 2024a; Abbasi et al., 2024b), neurodegenerative diseases (Kruczkowska et al., 2025), and cancers (Saengboonmee et al., 2017; Panaampon et al., 2023). Metformin’s mechanisms are not fully understood, but it is known to enhance insulin sensitivity, activate AMP-activated protein kinase (AMPK), inhibit mitochondrial respiratory complex I, and modulate gut microbiota. Additionally, it impacts lipid metabolism and inflammatory signaling, promotes autophagy, improves DNA repair, and reduces adipogenesis and adipokine secretion. Currently, metformin is a major focus of research beyond diabetes treatment, with many studies showing promising results already applied in clinical practice (Teede et al., 2023). This Research Topic compiles articles and reviews on the potential repurposing of metformin, aiming to deepen our understanding of its effects and benefits in various diseases.

This Research Topic presents seven original articles, including a systematic review, meta-analysis, and a brief research report. The studies cover preclinical models to randomized controlled trials, featuring a review and a perspective on metformin’s advancements. Metformin has shown real-world therapeutic efficacy in four clinical studies, including two randomized controlled trials. A pilot study by AlRasheed et al. found that Parkinson’s disease patients receiving levodopa/carbidopa along with metformin experienced significant improvements on the Unified Parkinson’s Disease Rating Scale (UPDRS) compared to their baseline scores. In contrast, those receiving only levodopa/carbidopa showed no significant changes. While final UPDRS scores were not significantly different between the groups, biomarkers in the metformin group indicated a trend toward positive outcomes. A double-blind, randomized, controlled trial by Binsaleh et al. found that adding metformin to standard treatment for mild to moderate ulcerative colitis may slow disease progression, as shown by a reduced disease activity index. A group of patients receiving metformin alongside mesalamine, a standard treatment for inflammatory bowel diseases, showed significantly decreased inflammation biomarkers and disease severity. This suggests metformin may have anti-inflammatory effects that could benefit other inflammatory conditions. Additionally, a clinical study reported that metformin concentrations affected plasma galectin-3 levels, a biomarker linked to polycystic ovary syndrome (PCOS), in patients preparing for *in vitro* fertilization, as noted by Nikolic et al. Metformin plasma concentration showed a weak correlation with galectin-3 levels, but its dosage was linked to plasma galectin-3 levels. This suggests that personalized metformin dosing is important for managing PCOS. Furthermore, another study indicated that metformin improved symptoms, knee joint function, and quality of life in individuals with impaired glucose tolerance and knee osteoarthritis (Halabitska et al.). Metformin is recommended for diabetes prevention in individuals with impaired glucose tolerance, and this study suggests that its early use may also benefit those without hyperglycemia. Patients with diabetes on metformin may not need to stop their medication before medical imaging with contrast agents. A systematic review and meta-analysis by Xu et al. found no significant impact of metformin on the risk of contrast-induced acute kidney injury, impaired renal function, or elevated lactate levels.

In addition to clinical studies, the present issue also delves into mechanistic studies using preclinical models, both *in vitro* and *in vivo* experiments. The anti-inflammatory effects of metformin were emphasized in a mouse model of neovascular age-related macular degeneration (AMD). The insight into the intracellular signaling of inflammation-mediated AMD was also reported in the same study by Wang et al. Mice with AMD that were treated with metformin showed significantly reduced retinal vascular leakage and neovascularization, as well as lower levels of inflammatory markers and phosphorylated STAT3, a key transcription factor involved in inflammation. However, it was noted that retinal fibrosis increased in mice receiving metformin, indicating that while metformin might be beneficial for specific stages of AMD, further studies are needed to explore this more thoroughly. The perspective of metformin’s roles, as well as other anti-diabetic drugs, on the prevention of AMD was also reported by Zhou and Xue in the present issue.

The AMPK-dependent effects of metformin are not only involved in anti-inflammation, but they also protect osteoblast cells from ferroptotic cell death under diabetic conditions. Using metformin treatment for patients with diabetes may help prevent diabetic osteoporosis by protecting osteoblasts, as shown by *in vitro* and *in vivo* experiments conducted by Liu et al. The results from this study are consistent with the clinical findings in the aforementioned article of Halabitska et al. Finally, the anti-cancer effects of metformin remain a hot issue in the present decade, as shown in the growing evidence in several cancers. The updated overview of metformin’s benefit in treating gynecological disorders, including cancers, is also included in this issue (Nie et al.).

This Research Topic enhances our understanding of metformin and its potential applications beyond diabetes treatment, suggesting it remains a strong candidate for drug repurposing. Already integrated into some clinical practices, metformin is the focus of extensive research from preclinical studies to clinical trials. Despite its long history and benefits for various conditions, the underlying molecular mechanisms of its effects have not been fully clarified (Foretz et al., 2023). Caution is advised in using metformin for age-related macular degeneration (AMD) treatment. A recent study suggests that while metformin may be beneficial, it could also increase the risk of retinal fibrosis in animal models. Furthermore, the pharmacokinetics of metformin in patients with diverse conditions is essential for its effective use. Future research on metformin’s molecular mechanisms and clinical benefits is expected to be important over the next decade, potentially broadening its medical applications (Zhou and Xue, 2025). This Research Topic issue could highlight the potential of repurposing drugs in drug discovery to improve human health and inspire further research into metformin and other drugs for treating various diseases.
